# Prevalence of and interventions for sarcopenia in ageing adults: a systematic review. Report of the International Sarcopenia Initiative (EWGSOP and IWGS)

**DOI:** 10.1093/ageing/afu115

**Published:** 2014-09-21

**Authors:** Alfonso J. Cruz-Jentoft, Francesco Landi, Stéphane M. Schneider, Clemente Zúñiga, Hidenori Arai, Yves Boirie, Liang-Kung Chen, Roger A. Fielding, Finbarr C. Martin, Jean-Pierre Michel, Cornel Sieber, Jeffrey R. Stout, Stephanie A. Studenski, Bruno Vellas, Jean Woo, Mauro Zamboni, Tommy Cederholm

**Affiliations:** 1 Servicio de Geriatría, Hospital Universitario Ramón y Cajal, Ctra. Colmenar km 9, 1, 28034 Madrid, Spain; 2 Istituto di Medicina Interna e Geriatria, Università Cattolica del Sacro Cuore, Rome, Italy; 3 Gastroentérologie et Nutrition Clinique, CHU de Nice, Université de Nice Sophia-Antipolis, Nice, France; 4 Universidad Autonoma de Baja California, Tijuana Baja California Mexico, Mexico; 5 Department of Human Health Sciences, Kyoto University, Graduate School of Medicine, Kyoto, Japan; 6 Unité de Nutrition Humaine, UMR 1019, INRA, Université Clermont-Ferrand, CHU de Clermont-Ferrand, France; 7 Center for Geriatrics and Gerontology, Taipei Veterans General Hospital, Taipei, Taiwan; 8 Nutrition, Exercise Physiology, and Sarcopenia Laboratory, Jean Mayer Human Nutrition Research Center on Aging at Tufts University, Boston, MA, USA; 9 Department of Ageing and Health, Guys and St Thomas' NHS Foundation Trust, London, UK; 10 Département de Réhabilitation et Gériatrie, Hôpitaux Universitaires de Genève-Suisse, Geneva, Switzerland; 11 Institut for Biomedicine of Ageing, University Erlangen-Nürnberg, Erlangen, Germany; 12 Institute for Exercise Physiology and Wellness Research, University of Central Florida, Orlando, FL, USA; 13 Division of Geriatric Medicine, University of Pittsburgh, Pittsburgh, PA, USA; 14 Department of Geriatric Medicine, Inserm U558 Le Centre Hospitalier Universitaire de Toulouse (CHU) – Gérontopôle, Toulouse, France; 15 Department of Medicine and Therapeutics, Prince of Wales, Hospital, Chinese University of Hong Kong, Hong Kong SAR, The People's Republic of China; 16 Division of Geriatrics, Department of Medicine, University of Verona, Verona, Italy; 17 Department of Public Health and Caring Sciences/Clinical Nutrition and Metabolism, Uppsala University, Uppsala, Sweden

**Keywords:** exercise intervention, nutrition intervention, prevalence, age-related, sarcopenia, older people

## Abstract

**Objective:** to examine the clinical evidence reporting the prevalence of sarcopenia and the effect of nutrition and exercise interventions from studies using the consensus definition of sarcopenia proposed by the European Working Group on Sarcopenia in Older People (EWGSOP).

**Methods:** PubMed and Dialog databases were searched (January 2000–October 2013) using pre-defined search terms. Prevalence studies and intervention studies investigating muscle mass plus strength or function outcome measures using the EWGSOP definition of sarcopenia, in well-defined populations of adults aged ≥50 years were selected.

**Results:** prevalence of sarcopenia was, with regional and age-related variations, 1–29% in community-dwelling populations, 14–33% in long-term care populations and 10% in the only acute hospital-care population examined. Moderate quality evidence suggests that exercise interventions improve muscle strength and physical performance. The results of nutrition interventions are equivocal due to the low number of studies and heterogeneous study design. Essential amino acid (EAA) supplements, including ∼2.5 g of leucine, and β-hydroxy β-methylbutyric acid (HMB) supplements, show some effects in improving muscle mass and function parameters. Protein supplements have not shown consistent benefits on muscle mass and function.

**Conclusion:** prevalence of sarcopenia is substantial in most geriatric settings. Well-designed, standardised studies evaluating exercise or nutrition interventions are needed before treatment guidelines can be developed. Physicians should screen for sarcopenia in both community and geriatric settings, with diagnosis based on muscle mass and function. Supervised resistance exercise is recommended for individuals with sarcopenia. EAA (with leucine) and HMB may improve muscle outcomes.

## Introduction

Although exercise and nutrition interventions have proved efficacy to treat different conditions in various populations of adults and older people, the effects in those with sarcopenia have received less attention. Sarcopenia has been defined as the loss of skeletal muscle mass and strength that occurs with advancing age [**1**]. However, until recently, there has been no widely accepted definition of sarcopenia that was suitable for use in research and clinical practice.

A practical clinical definition of, and consensus diagnostic criteria for, age-related sarcopenia was developed in 2009–10 and reported by the European Working Group on Sarcopenia in Older People (EWGSOP) [2]. The EWGSOP provided a working definition of sarcopenia as ‘a syndrome characterised by progressive and generalised loss of skeletal muscle mass and strength with a risk of adverse outcomes such as physical disability, poor quality of life and death’ [2]. They proposed that sarcopenia is diagnosed using the criteria of low muscle mass and low muscle function (either low strength and/or low physical performance) [2]. A similar approach was taken in 2009 by the International Working Group on Sarcopenia (IWGS), who provided a consensus definition of sarcopenia as ‘age-associated loss of skeletal muscle mass and function’. This group proposed that sarcopenia is diagnosed based on a low whole-body or appendicular fat-free mass in combination with poor physical functioning [**3**].

To date, most prevalence and intervention studies have used varied definitions of sarcopenia that are not current (e.g. based only on decreased muscle mass) and the results may therefore be misleading and difficult to interpret. However, with the implementation of new operational definitions of sarcopenia, it may be possible to define the natural course of the condition and determine which treatments are effective. In 2013, representatives of the EWGSOP, IWGS and international experts from Asia and America came together to form the International Sarcopenia Initiative (ISI) with the intention of developing a systematic review of some aspects of sarcopenia. Specifically, the aims of this systematic review were to (i) assess the prevalence of sarcopenia using definitions that include both muscle mass and muscle function, as proposed by the EWGSOP and the IWGS; and (ii) to review interventions with nutrition and exercise that used both muscle mass and muscle function as outcomes.

## Methods

### Search strategy

**Figure 1.**
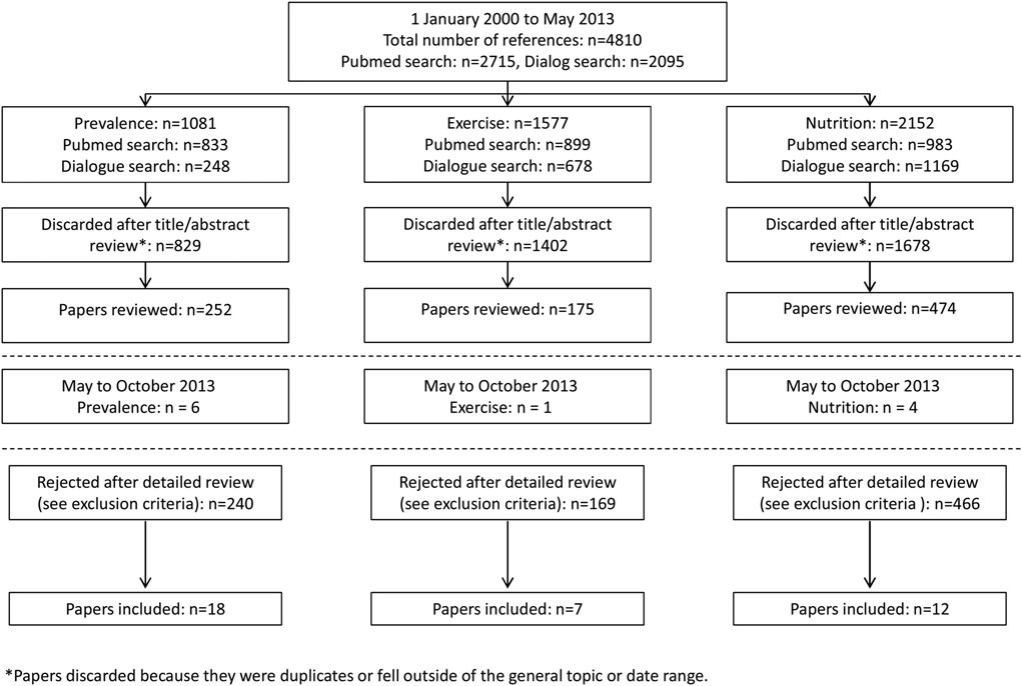
Selection of papers.

PubMed and Dialog databases were searched from January 2000 to May 2013 using the pre-defined search terms sarcopenia and muscle mass: additional pre-defined search terms were applied (see Supplementary data available in *Age and Ageing* online, Appendix S1) for each of the three areas of interest: prevalence of sarcopenia, nutrition interventions for sarcopenia and exercise interventions for sarcopenia (Figure 1). An additional short search of PubMed and Dialog databases using the terms ‘sarcopenia’, ‘elderly’, ‘intervention’, ‘prevalence’ and ‘treatment’ was conducted to cover articles published in the period May–October 2013 (Figure 1). The reference lists of systematic review articles and meta-analyses were scanned for any additional references missed from the PubMed and Dialog searches. The expert group was also asked to identify and provide any additional papers; they deemed to have been missed in the formal literature searches.

### Eligibility criteria

Across all three categories, only studies that enrolled participants aged 50 years and older within well-defined populations (such as those in community-dwelling, hospital and nursing home/geriatric settings) were included. Prevalence studies were included if sarcopenia had been assessed according to the EWGSOP definition of sarcopenia, i.e. based on muscle mass *and* muscle strength *or* physical performance [2]. They were excluded if they only used muscle mass to define sarcopenia. Nutrition and exercise intervention studies were included if the outcome measures reported for the interventions included muscle mass and at least one measure of muscle strength or physical performance, even when the population studied was not defined as sarcopenic. If these outcomes were not clearly stated within the study methodology, the study was excluded. Other criteria used to exclude studies in each of the three categories are provided in Supplementary data available in *Age and Ageing* online, Appendix S2.

Observational studies were included in the prevalence category, but for the exercise and nutrition intervention categories, only randomised controlled trials were selected. The ISI group was divided into three subgroups (prevalence, exercise and nutrition). Final papers selected for inclusion in each of the three categories were agreed upon by each subgroup consensus.

### Data synthesis

Data tables were compiled independently for each topic. For the prevalence of sarcopenia category, data were recorded on demographics (country, gender and age), assessment method used for each domain (muscle mass, muscle strength and physical performance) and sarcopenia prevalence. For the interventional categories, data were collected on population, numbers studied (by gender), age, intervention, control group, duration of intervention, outcomes measured and the main results. The methodological quality of each randomised, controlled trial was assessed using the 11-point Physiotherapy Evidence Database (PEDro) scale. Each item on the scale that the trial satisfied (except for item 1, which assesses external validity and is not included in the total score) contributed one point to the total PEDro score, with 0 representing the lowest score and 10 the highest [4]. This scale was specifically developed to rate the quality of randomised, controlled trials evaluating physical therapist interventions.

The following questions were investigated in patients aged 50 years and older without comorbid conditions. What is the prevalence of sarcopenia in different populations? Is physical exercise (as physical activity, resistance training or endurance training) effective compared with control in improving measures of muscle loss, muscle mass, muscle strength and physical performance? Compared with control, does nutrition supplementation improve measures of muscle mass, muscle strength, and physical performance? Based on the answers to these questions, draft recommendations were proposed by the co-chairs, and the working group then reviewed these recommendations to reach a consensus.

## Results

Overall, 4810 publications were identified (Figure 1). Of these, 3909 were excluded, leaving 901 publications for potential inclusion (prevalence: 252; exercise: 175; nutrition: 474). In addition, 11 papers were identified as suitable for inclusion as a result of a short search of PubMed and Dialog databases to identify articles published in the period May–October 2013.

Eighteen prevalence, 7 exercise and 12 nutrition papers were finally chosen by the working group members for inclusion within this review (Figure 1).

### Estimates of prevalence

Of the 18 prevalence studies meeting the inclusion criteria, 15 (83%) were in community-dwelling patients [5, **6–9**, 10, 11, **12, 13**, 14, 15, **16**, 17, **18**, 19], with two studies in patients in long-term care institutions [**20, 21**], and one publication in the acute hospital-care setting [**22**] (Table 1). The reporting of age varied across studies, but for those where the mean age was given, this ranged from 59.2 to 85.8 years [5, **6–9**, 10, 11, **12, 13**, 14, **16**, 17, **18**, 19, **21**].

**Table 1.** Prevalence of sarcopenia

Reference	Date data collected	Country	M/F, *n*	Assessment method			Age, years Mean (SD) [Range]	Sarcopenia prevalence, %		
				Muscle mass	Muscle strength	Physical performance		Total	Male	Female
Community-dwelling populations										
Abellan van Kan *et al.* [5]	Jan 1992–Jan 1994	France	0/3025	DEXA	HS	GS	80.51 (3.9) [≥75]	5.2	–	5.2
Landi *et al.* [**6**]	Oct 2003	Italy	66/131	MAMC	HS	GS	82.2 (1.4) [80–85]	21.8	25.7	19.8
Landi *et al.* [**7**]	Oct 2003	Italy	118/236	MAMC	HS	GS	85.8 (4.9)	29.1	27.1	30.1
Lee *et al.* [**8**]	–	Taiwan	223/163	DXA	HS, KE, PEF	SPPB, GS, TUG, or SCPT	73.7 (5.6)	7.8^a^ 16.6^b^	10.8^a^ 14.9^b^	3.7^a^ 19.0^b^
Legrand *et al.* [**9**]	Nov 2008–Sep 2009	Belgium	103/185	BIA	HS	mSPPB, GS	84.8 (3.6) [>80]	12.5	14.6	12.4
Malmstrom *et al.* [10]	Sep 2000–Jul 2001	USA (African Americans)	124/195	DEXA	–	GS	59.2 (4.4)	4.1	–	–
McIntosh *et al.* [11]	–	Canada	42/43	BIA	HS	GS	75.2 (5.7)	6.0	S: 5 SS: 0	S: 7 SS: 0
Murphy *et al.* [**12**]	–	USA	1426/1502	DEXA	HS	GS	F: 73.5 (2.88) M: 73.8 (2.85) Total: [70–79]	S: 5	–	–
Patel *et al.* [**13**]	–	UK^c^	Cohort A: 103/0 Cohort B: 765/1022	DEXA, SFT	HS	GS, TUG, chair-rise time	(A): 72.5 (2.5) (B): M, 67.0 (2.6); F, 67.1 (2.6)	(A): 6.8 (B): 7.8	4.6	7.9
Patil *et al.* [14]	–	Finland	0/409	DEXA	HS	GS, SPPB, TUG	74.2 (3.0) [70–80]	0.9	–	0.9
Sanada *et al.* [15]	–	Japan	0/533	DEXA	HS, LEP	Sit and reach, VO_2max_	<39: 11.4% <49: 21.2% <59: 25.9% <69: 29.8% <85: 11.6% [30–84]	24.2	–	24.2
Tanimoto *et al.* [**16**]	May–Jun 2007, 2008, 2009	Japan	364/794	BIA	HS	GS	M: 74.4 (6.4) F: 73.9 (6.3) [≥65]	–	11.3	10.7
Verschueren *et al.* [17]	–	Belgium, UK	679/0	DEXA	HS, KE	GS	59.6 (10.7) [40–79]	S: 3.7 SS: 0	–	–
Volpato *et al.* [**18**]	2004–2006	Italy	250/288	BIA	HS	GS	77.1 (5.5) [65–97]	10.2	2.6	6.7
Yamada *et al.* [19]	–	Japan	568/1314	BIA	HS	GS	74.9 (5.5) [65–89]	–	21.8	22.1
Institutional dwelling										
Bastiaanse *et al.* [**20**]	–	Netherlands	450/434	CC	HS	GS	50–59: 46.5% 60–69: 35.2% 70–79: 16.2% ≥80: 2.1% [≥50]	All: 14.3 50–64: 12.7 ≥65: 17.4	–	–
Landi *et al.* [**21**]	Aug–Sep 2010	Italy	31/91	BIA	HS	GS	84.1 (4.8) [≥70]	32.8	67.7	20.8*
Acute hospital care										
Gariballa and Alessa [**22**]	–	UK	227/205	MAMC	HS	–	[≥65]	10.2	–	–

ALM, appendicular lean mass; BIA, bioelectrical impedance analysis; CC, calf circumference; DEXA, dual-energy X-ray absorptiometry; F, female; GS, gait speed; HS, hand-grip strength using a dynamometer; KE, knee extensor; LEP, leg extension power; M, male; MAMC, mid-arm muscle circumference; PEF, peak expiratory flow; S, sarcopenia; SCPT, stair-climb power test; SD, standard deviation; SFT, skin-fold thickness; (m)SPPB, (modified) standard physical performance battery; SS, severe sarcopenia; TUG, timed-up-and-go; VO_2max_, maximal oxygen uptake.

^a^By relative appendicular skeletal muscle index.

^b^By percentage skeletal muscle index.

^c^Consists of two cohorts (Cohort A: detailed data were collected. Cohort B: same data were collected, but no DEXA).

* *P* < 0.001 versus females.

The prevalence of EWGSOP-defined sarcopenia was 1–29% (up to 30% in women) for older adults living in the community [5, **6–9**, 10, 11, **12, 13**, 14, 15, **16**, 17, **18**, 19], 14–33% (up to 68% in men) for those living in long-term care institutions [**20, 21**] and 10% for those in acute hospital care [**22**]. Age was not consistently reported across the studies, with some giving mean ages only, others reporting ranges, and others breaking age down into categories; thus, a comprehensive analysis of prevalence based on age could not be made. However, where reported, the majority of studies suggested the prevalence of sarcopenia increased with age [**18**, 19, **22**]. However, one study appeared to show a decrease in sarcopenia prevalence with increasing age [**20**]. In one study, sarcopenia appeared to be related to gender, with males more commonly affected than females [**21**], while another study showed a numerically higher prevalence of sarcopenia and severe sarcopenia in women than in men [**13**]. In a further study, the prevalence of sarcopenia was higher in women than in men in those aged <75 years; but, in those aged >85 years, the prevalence of sarcopenia was higher in men than in women (*P* < 0.05) [19]. However, in most studies that reported gender, there was no significant association with sarcopenia prevalence [**6–9**, 11, **16**, 19, **20**].

### Exercise interventions

There were seven moderate quality (PEDro score: 4–6) intervention studies that investigated the effect of exercise on muscle parameters in different populations aged 60–95 years (Table 2) [**23–29**]. The impact of exercise on sarcopenia was assessed using muscle mass and muscle strength or power measures in all studies [**23–29**]; assessment of physical performance (chair rise [**24**], 12-min walk [**25**], stair climbing [**29**] or timed up and go [**27, 28**]) was carried out in five of seven studies (Table 2).

**Table 2.** Summary of the effect of exercise on sarcopenia in randomised, controlled studies meeting the inclusion criteria

Reference	Population	Number studied (M/F)	Age, years Mean (SD) [Range]	Intervention		PEDro score	Outcomes measured	Main results
				Description	Duration (months)			
Binder *et al.* [**23**]	Frail, community-dwelling	91	83 (4)	Progressive RET; CON (low-intensity home exercise)	9	5	MM (DEXA), MS (KE)	Total body FFM increased in the progressive RET group, but not in the CON group (*P* = 0.005) MS increased to a greater extent in the progressive RET than in the CON group (*P* = 0.05)
Bonnefoy *et al.* [**24**]	Frail, care institution	57 (7/50)	83	RET + SUPP; CON + SUPP; RET + PLA; PLA + CON	9	5	MM (FFM by labelled water), MP, PP (chair rise)	RET did not improve MM or MP, but improved PP versus CON (*P* = 0.01)
Bunout *et al.* [**25**]	Community-dwelling	98 (36/62)	≥70	RET + SUPP; SUPP; RET; CON	18	4	MM (DEXA), MS (quadriceps strength), PP (12-min walk)	FFM did not change in any group RET improved MS versus CON (*P* < 0.01) PP remained constant in RET group, but declined in the CON group (*P* < 0.01).
Suetta *et al.* [**29**]	Frail, post-operative elective hip replacement	36 (18/18)	[60–86]	RET; ES; CON (standard rehabilitation)	3	5	MM (US), MS (quadriceps), PP (stair climbing)	RET improved MM, MS and PP versus CON (all *P* < 0.05) In the ES or CON groups, there was no increase in any measurement outcomes
Goodpaster *et al.* [**26**]	Sedentary, community-dwelling	42 (11/31)	[70–89]	PA (aerobic, strength, flexibility, balance training); CON (health education)	12	5	MM (CT scan), MS (KE)	MM decreased in both groups (but losses were not different between groups) MS loss was decreased in CON, but completely prevented in PA (between group change not significant)
Kemmler *et al.* [**27**]	Community-dwelling	246 (0/246)	69.1 [65–80]	High-intensity multipurpose exercise programme; CON (wellbeing)	18	6	MM (DEXA), MS (isometric leg extension), PP (timed up and go)	Multipurpose exercise was associated with significant improvements in MM (*P* = 0.008), MS (*P* = 0.001), PP (*P* < 0.001) versus CON
Rydwik *et al.* [**28**]	Frail, community-dwelling	96 (38/58)	>75	PA (aerobic, muscle strength, balance exercises); nutrition intervention; PA + nutrition intervention; CON	3	5	MM [FFM = BW-fat mass (skin folds)], MS (leg press, dips), PP (timed up and go)	PA improved MS (*P* < 0.01 for dips), but did not improve MM or PP versus CON

BW, body weight; CON, control; CT, computerised tomography; DEXA, dual-energy X-ray absorptiometry; ES, electrical stimulation; F, female; FFM, free-fat mass; FM, fat mass; KE, knee extension; M, male; min, minute; MM, muscle mass; MP, muscle power; MS, muscle strength; RET, resistance exercise training; PA, physical activity; PLA, placebo; PP, physical performance; SD, standard deviation; SUPP, nutritional supplement; US, ultrasound.

#### Resistance training interventions

Resistance training was explored in four mixed-gender studies (Table 2) [**23–25, 29**]. When used from 3–18 months, resistance training interventions alone improved muscle mass in two of four studies [**23, 29**] and muscle strength in three of four studies [**23, 25, 29**] compared with control (low-intensity home exercise or standard rehabilitation). Physical performance (chair rise, stair climb or 12-min walk) improved with resistance training alone versus control in all three studies assessing this parameter [**24, 25, 29**].

#### Combined exercise/physical activity interventions

Three additional studies explored compound exercise interventions (with different blends of aerobic, resistance, flexibility and/or balance training), which were performed for 3–18 months [**26–28**]. A high-intensity multipurpose exercise programme over 18 months improved muscle mass, muscle strength and physical performance versus control (wellbeing) in a study in 246 women [**27**]. In two mixed-gender studies [**26, 28**], muscle mass did not improve; muscle strength (assessed as dips) improved with physical activity versus control at 3-months follow-up in one of the two studies [**28**]; and physical performance did not improve in the one study in which it was assessed [**28**].

Overall, most exercise trials showed improved muscle strength and physical performance (using different measures), but only three of seven studies found increased muscle mass. These trials were largely performed in community-dwelling older people, sometimes identified as frail by different measures.

### Nutrition interventions

Most studies (11/12) evaluating nutrition intervention in adults aged 50 years and over (Table 3) were in community-dwelling populations whose age ranged from 62 to 90 years (*n* = 14–98) [**25, 30–39**]. One study assessed individuals living in care institutions (mean age, 83 years; *n* = 57) [**24**]. Nutrition interventions that were identified included protein supplementation (usually with other nutrients providing extra calories) [**24, 25, 30, 37, 38**], amino acid (mainly leucine) supplementation [**33, 35**], β-hydroxy β-methylbutyric acid (HMB; a bioactive metabolite of leucine) supplementation with arginine [**34**] or alone [**32, 34, 36, 39**] or fatty acid supplementation [**31**] administered over 8–36 weeks to evaluate changes in muscle mass and/or strength and function.

**Table 3.** Summary of the effect of nutrition on sarcopenia in randomised, controlled studies meeting the inclusion criteria

Reference	Population	Number studied (M/F)	Age, years, mean (SD) [range]	PEDro Score	Intervention (duration)	Outcomes measured	Main results
Bonnefoy *et al.* [**24**]	Frail, care institution	57 (7/50)	83	5	RET + SUPP (400 kcal, 30 g of protein/day); CON + SUPP; RET + PLA; PLA + CON (9 months)	MM (FFM by labelled water), MP, PP (chair rise, 6-min walk, stair climb)	SUPP significantly increased MP at 3 months versus CON (*P* = 0.03), but not at 9 months SUPP did not improve MM or PP versus CON
Bunout *et al.* [**25**]	Community-dwelling	98 (36/62)	[≥70]	4	RET + SUPP (400 kcal, 13 g of protein/day); SUPP; RET; CON (18 months)	MM (DEXA), MS (biceps and quadriceps strength), PP (12-min walk)	SUPP alone had no effect on MM, MS or PP SUPP did not show an additive effect over RET outcome
Chale *et al.* [**30**]	Sedentary, community-dwelling	80 (33/47)	[70–85]	10	WPS (378 kcal, 40 g of protein/day) + RET; CON (378 kcal, no protein) + RET (6 months)	MM (DEXA, CT scan), MS (KE), PPPP (stair climb, chair rise, 400 m walk, SPPB)	WPS + RET did not improve MM, MS or PP significantly versus CON + RET
Tieland *et al.* [**37**]	Frail, community-dwelling	62 (21/41)	PLA: 79 (6) Protein: 78 (9) [≥65]	10	Protein (30 g/day) + RET; PLA + RET (24 weeks)	MM (DEXA), MS (leg press, LE, HS), PP (SPPB)	Protein + RET significantly improved MM (*P* = 0.006), but not MS or PP versus PLA + RET
Tieland *et al.* [**38**]	Frail, community-dwelling	65 (29/36)	PLA: 81 (±1 SEM) Protein 78 (±1 SEM) ≥65	8	Protein (30 g/day); PLA; (24 weeks)	MM (DEXA), MS (leg press, LE, HS), PP (SPPB)	PP improved significantly with protein supplementation (*P* = 0.02), but not MM or MS versus PLA
Dillon *et al.* [**33**]	Healthy individuals	14 (0/14)	All: 68 (±2) PLA: 69 (±3) Supplement: 67 (±1)	7	EAA (HIS, ILE, LEU, LYS, MET, PHE, THR, VAL); PLA; (3 months)	MM (DEXA), MS (bicep curl, triceps extension, LE, leg curl)	EAA increased MM versus baseline, (*P* < 0.05) There were no changes in MS
Kim *et al.* [**35**]	Community-dwelling	155 (0/155)	79 (2.9) [≥75]	8	EAA (LEU, LYS, VAL, ILE, THR, PHE) + RET; EAA; RET; HE (3 months)	MM (BIA), MS (KE), PP (max. walking speed)	EAA alone improved PP, but not MM and MS versus HE EAA + RET improved leg (not appendicular or total) MM (*P* < 0.007) and, MS (*P* = 0.02) versus HE PP was not more improved by the addition of EAA than by RET alone
Flakoll *et al.* [**34**]	Community-dwelling	57 (0/57)	76.7 [62–90]	8	ARG + HMB + LYS; PLA (12 weeks)	MM (BIA), MS (isometric leg strength, HS), PP (get up and go)	MS (*P* ≤ 0.05) and PP (*P* = 0.002) significantly improved with ARG + HMB + LYS versus PLA ARG + HMB + LYS did not significantly improve MM versus PLA
Deutz *et al.* [**32**]	Healthy individuals on bed rest	19 (4/15)	PLA: 67.1 (±1.7) HMB: 67.4 (±1.4) [60–76]	10	HMB; PLA Bed rest (10 days) + rehabilitation (8 weeks)	MM (DEXA), MS (KE, leg press), PP (SPPB, get up and go, 5-item PPB)	Bed rest caused a significant decrease in MM (*P* = 0.02) in the PLA group, but MM was preserved in the HMB group Changes in MS and PP were not significant for HMB versus PLA
Stout *et al.* [**36**]	Community-dwelling	98 (49/49)	73 (±1 SEM) [≥65]	9	Phase I: HMB; PLA (24 weeks) Phase II: PLA + RET; HMB + RET (24 weeks)	MM (DEXA), MS (isokinetic leg strength, HS), PP (get up and go)	HMB alone significantly improved some, but not all measures of MS versus PLA. No significant changes were found in MM and PP with HMB versus PLA Adding HMB to RET did not improve any parameters over RET alone
Vukovich *et al.* [**39**]	Community-dwelling	31 (15/16)	70 (±1)	10	HMB + RET; PLA + RET (8 weeks)	MM (DEXA, CT scan), MS (misc. upper and lower body strength press, flexion and extension measurements)	MM improved with HMB + RET versus PLA + RET, but not significantly (*P* = 0.08) MS did not improve with HMB + RET versus PLA + RET
Cornish and Chilibeck [**31**]	Community-dwelling	51 (28/23)	65.4 (±0.8)	10	ALA + RET; PLA + RET (12 weeks)	MM (DEXA, US), MS (leg press, chest press)	ALA + RET had minimal effect on MM or MS versus PLA + RET

ALA, α-linolenic acid; ARG, arginine; BIA, bioelectrical impedance analysis; CON, controls; CT, computerised tomography; DEXA, dual X-ray absorptiometry; EAA, essential amino acid; F, female; FFM, fat-free mass; HE, health education; HIS, histidine; HMB, β-hydroxy β-methylbutyrate; ILE, isoleucine; HS, hand-grip strength; KE, knee extension; LE, leg extension; LEU, leucine; LYS, lysine; M, male; min, minute; MET, methionine; MM, muscle mass; MP, muscle power; MS, muscle strength; NS, not significant; PHE, phenylalanine; PLA, placebo; PP, physical performance; RET, resistance exercise training; SD, standard deviation; SPPB, standard physical performance battery; SUPP, nutritional supplement; THR, threonine; VAL, valine; WPS, whey protein supplement.

#### Protein supplements

Protein supplementation (with other nutrients providing ∼400 extra kilocalories/day in three of five studies) either alone or in addition to resistance exercise training was evaluated in five moderate- to high-quality (PEDro score: 4–10) studies [**24, 25, 30, 37, 38**]. In the only high-quality study with no associated exercise in a frail, community-dwelling population, protein supplementation improved physical performance, but not muscle mass or muscle strength versus control [**38**]. Only in one of the four moderate- to high-quality studies using different types and amounts of protein supplementation in addition to an exercise programme for 24 weeks to 18 months [**24, 25, 30, 37**], was muscle mass increased over the control group [**40**]. Muscle strength did not change in any of the studies; only a transient increase in muscle power was found in one study [**24**]. Physical performance did not improve in any of these four studies.

Overall, these five moderate- to high-quality studies fail to show a consistent effect of protein supplementation on muscle mass and function [**24, 25, 30, 37, 38**].

#### Essential amino acid supplementation

The effect of essential amino acid (EAA) supplementation either alone [**33**] or in combination with resistance exercise training [**35**] on muscle parameters was investigated in two high-quality (PEDro score: 7 and 8) studies of 3 month's duration each, in community-dwelling individuals. Daily leucine amount provided was 2.8 and 2.5 g. EAA improved muscle mass in one of two studies [**33**], did not improve muscle strength, and improved physical performance in the study that used this outcome [**35**]. When combined with exercise, EAA improved leg muscle mass and muscle strength but not physical performance versus health education at 3 months [**35**].

Overall, very limited evidence on EAA supplementation seems to show some effects on muscle mass and function.

#### HMB supplementation

The effect of HMB alone [**32, 36**] or HMB in combination with ARG and LYS [**34**] or resistance exercise training [**39**] on muscle parameters has been investigated in four high-quality (PEDro score: 8–10) studies of 8–24-week duration in community-dwelling older adults [**34, 36, 39**] or in healthy older adults on extended bed rest [**32**]. HMB prevented muscle mass loss in one of four studies and did not improve muscle mass in the other three [**32**]; improved muscle strength in one [**34**] (and possibly two) [**36**] of four studies and improved physical performance in one of four studies [**34**].

Overall, HMB showed some effects on muscle mass and function in these high-quality studies, but sample sizes were small.

#### Fatty acids

The only study examining the effect of fatty acid supplementation (α-linolenic acid) on muscle parameters (PEDro score: 10), in 51 older adults undergoing resistance training for 12 weeks, showed no effect of the supplementation on muscle mass or muscle strength versus placebo [**31**].

## Discussion

Sarcopenia is an independent risk factor for adverse outcomes, including difficulties in instrumental and basic ADL [**6**, 10, **16, 20, 21**], osteoporosis [17], falls [**21**], hospital length of stay and re-admission [**22**] and death [**6**]. This underscores the importance of understanding the true prevalence of sarcopenia and effective preventative strategies.

### Prevalence

The prevalence of sarcopenia in the literature varies widely, and is likely to be affected by the population studied (including the population under investigation and the reference population) and the different methods used to assess muscle mass, muscle strength and physical performance [**3**]; although results may also be due to real differences in prevalence of sarcopenia. As studies that defined sarcopenia as muscle mass plus muscle strength/physical performance were few, comparisons of prevalence across studies were difficult due to the different methods and cut-off points used. The prevalence of sarcopenia in the community using a definition consistent with EWGSOP was 1–33% across different populations (male and female data combined), with higher prevalence, as expected, in settings where older, more complex or acutely ill individuals are cared for. Ethnicity may also play a role, especially if the reference and study populations do not match.

After careful consideration of the methodological limitations and scope of these studies, the ISI group proposes certain recommendations for the design of future studies (expert advice):

- Studies with sufficient sample size to identify prevalence and risk factors for sarcopenia, including subpopulation analyses, are needed.
- Studies should focus on standardised, well-defined, reproducible populations, namely community-dwelling individuals, individuals living in nursing homes/care homes, and acutely ill or physically frail inpatients. These populations should be clearly described so that studies can be compared for external validity.
- Standardised models and cut-off points should be used for each domain of the definition of sarcopenia to allow comparison between studies.
- Longitudinal studies on the incidence of sarcopenia are needed, again using standard methods.

### Exercise intervention

Exercise interventions appear to have a role in increasing muscle strength and improving physical performance, although they do not seem to consistently increase muscle mass, in frail, sedentary, community-dwelling older individuals. Investigations in other populations are still anecdotal. No trials were found that recruited individuals based on their sarcopenic status. The results suggested that combining various types of exercise into a programme may also improve muscle strength and physical performance. Most exercise studies involved limited participants and were mainly conducted within a single country.

Recommendations for the design of exercise studies (expert advice):

- Improved standardisation of exercise interventions is needed, to allow for replication and contrast.
- Studies should have common outcome measures, along with similar time points for assessment (e.g. 4 weeks, 8 weeks, 3 months, 6 months, 1 year), so that valid comparisons across studies can be made. The short physical performance battery, gait speed, 400-m walking distance and grip strength are proposed as useful measures of physical performance that are able to determine clinically significant changes. Grip strength, chair rise and knee extension may be used to measure muscle strength.
- Exercise interventions should focus on well-defined populations, with well-defined sarcopenia.

### Nutrition intervention

Although nutrition intervention is considered one of the mainstays of intervention in sarcopenia, much of the evidence is based on short-term protein synthesis studies, and large clinical trials are still lacking. Our review has failed to show a consistent effect of protein supplementation, although the number of studies found using our strict selection criteria was very low. EAAs (with ∼2.5 g of leucine) and HMB seem to have some effects on muscle mass and muscle function that need to be confirmed in larger trials. Vitamin D studies were evaluated as part of the review process; while some epidemiological studies link vitamin D levels with muscle parameters, there were no intervention studies meeting the criteria for inclusion in this review. Similarly, there is a large literature on the effects of omega 3-fatty acids on muscle parameters, especially in cachexia, but only one negative study was found in this review [**31**]. Interventions that evaluated the combined effects of exercise and nutrition sometimes suggested a potential additive effect, although this needs further research. However, solid evidence on which to base recommendations for patients with sarcopenia is not available.

#### Recommendations for the design of nutrition studies (expert advice)

- Further studies are needed to determine the effect of different nutrition interventions on muscle mass and function using robust, multi-centre and standardised approaches with single or complex nutrition interventions and clinically relevant outcomes (muscle strength, physical performance).
- Studies using four arms (exercise, nutrition, both or none) should also be conducted. The choice of exercise and nutrition interventions should be based on the singular effect of each intervention.
- Outcome measures for such studies should not differ from those used for individual components, and reporting should allow for individual group comparisons to also evaluate the role of each component.
- Timing of nutrition intervention before or after exercise should be explored in clinical trials comparing different times of administration, as basic studies suggest there may be time-associated differences in the effect of nutrition intervention over exercise.
- Baseline nutritional status and physical frailty of the population should be considered when doing nutrition intervention studies.

### Practice recommendations

Sarcopenia is a common clinical problem in people over 50 years of age, and one that leads to severe adverse outcomes. Research on management interventions is advancing quickly, but questions still remain. Based on our current understanding, the expert group agreed some general recommendations for clinical practice (expert opinion):

1. Sarcopenia, defined as low muscle mass and low muscle function and/or reduced physical performance, occurs in at least 1 in 20 community-dwelling individuals, and prevalence may be as high as 1 in 3 in frail older people living in nursing homes (Table 1).
   • Owing to the consequences of sarcopenia on quality of life, disability and mortality, it is recommended that physicians should consider screening for sarcopenia, both in community and geriatric settings.
   • The new definitions of sarcopenia, based on muscle mass and function, should be preferred to definitions based on muscle mass alone.
2. Exercise interventions, especially those based on resistance training, may have a role in improving muscle strength and physical performance (moderate quality evidence), but not muscle mass. Moreover, exercise has been shown to improve other common conditions in adults and older patients, as well as being safe.
   • Supervised resistance exercise or composite exercise programmes may be recommended for frail or sedentary community-dwelling individuals.
   • Time of intervention of at least 3 months and probably longer may be needed to obtain significant improvement in relevant clinical parameters (muscle strength and physical performance). Increased physical activity in daily life may also be recommended in these individuals.
3. Some nutrition interventions such as EAAs (with ∼2.5 g of leucine) and HMB may improve muscle parameters. Although our findings did not appear to support this approach, increasing protein intake to 1.2 g/kg body weight/day, either by improving diet or adding protein supplements, has been recommended for adults and older people by an expert group [**40**]. Evidence to recommend specific interventions is yet to be established.

**Key points**

- The reported prevalence of sarcopenia in the community is up to 33%, with higher prevalence in long-term and acute care settings.
- This underscores the importance of preventative and clinical management strategies for managing sarcopenia.
- While further research is needed on interventions, we provide recommendations for clinical practice.
- The ISI included representatives of the European Working Group on Sarcopenia in Older People (EWGSOP), the International Working Group on Sarcopenia (IWGS) and international experts.

## Conflicts of interest

Abbott had no role in the choice of members of the group, but had the right to have an observer member at the meetings. Members of the Working Group received no salary or other incomes from the European Union Geriatric medicine Society (EUGMS), Abbott Nutrition (AN) or any other organisation for any of the tasks involved in the preparation of this manuscript or for attending the meetings of the group. An individual COI form has been filled by each member of the International Sarcopenia Initiative group. Medical writing support was provided by Mike Musialowski at Lucid with funding from AN.

## Funding

This work was supported by an unrestricted educational grant provided by AN to EUGMS. This grant was used for operational activities including two meetings of the Working Group.

## Supplementary data

Supplementary data mentioned in the text is available to subscribers in *Age and Ageing* online.

Supplementary Data
