# Supplementary material for: Prevalence of and interventions for sarcopenia in ageing adults: a systematic review. Report of the International Sarcopenia Initiative (EWGSOP and IWGS)
Source: Age Ageing. 2014 Sep 21;43(6):748–59. doi: 10.1093/ageing/afu115 (PMC4204661; doi:10.1093/ageing/afu115)
Supplement: Supplementary Data [file supp_43_6_748__index.html]

Prevalence of and interventions for sarcopenia in ageing adults: a systematic review. Report of the International Sarcopenia Initiative (EWGSOP and IWGS) — Prevalence of and interventions for sarcopenia in ageing adults: a systematic review. Report of the International Sarcopenia Initiative (EWGSOP and IWGS) — Supplementary Data 

# Prevalence of and interventions for sarcopenia in ageing adults: a systematic review. Report of the International Sarcopenia Initiative (EWGSOP and IWGS)

## Supplementary Data

Supplementary Data

**Files in this Data Supplement:**

- Supplementary Data - Docx file
