# Supplementary material for: Prevalence of and interventions for sarcopenia in ageing adults: a systematic review. Report of the International Sarcopenia Initiative (EWGSOP and IWGS)
Source: Age Ageing. 2014 Sep 21;43(6):748–59. doi: 10.1093/ageing/afu115 (PMC4204661; doi:10.1093/ageing/afu115)
Supplement: Supplementary Data [file supp_afu115_afu115supp.docx]

**Supplementary Data**

**Appendix 1**

**Methods**

Search terms for the prevalence and incidence of sarcopenia were: muscle wasting, age-related muscle loss, myopenia, dynapenia, prevalence, incidence, human, aged (excluding: cancer, obesity, stroke, heart failure, renal failure, chronic obstructive), and muscle strength, muscle mass, muscle function, frailty. The terms searched for exercise interventions were: physical exercise, physical activity, sarcopenia, muscle mass, lean body mass, fat-free mass, aging. Finally, those for nutrition interventions were: sarcopenia, protein, nutrition, supplement, lipid, fatty acid, amino acid, omega, β-hydroxy β-methylbutyrate (HMB), creatine, vitamin D, leucine, insulin, frailty, protein synthesis, muscle mass, muscle strength, lean body mass, fat-free mass, physical, performance, function, death, survival, mortality, longevity, lifespan (excluding mouse), disability, aging. The search strings were refined for each database to conform to the appropriate syntax and searching strategy required. There were no language limitations.

Some studies were excluded because of their primary focus (e.g. animal studies or those with a focus on genetics, biochemistry, biomarkers, or endocrinology), whereas others were excluded if patients had serious co-morbidities (e.g. cancer, or renal, hepatic, cardiac or respiratory diseases). All exclusion criteria are given in Appendix 2, Table 1.

**Appendix 2**

**Results**

**Table 1. Exclusion criteria based on the principal focus of the study and non-sarcopenia diseases present.**

| **Prevalence** | **Exercise** | **Nutrition** |
| --- | --- | --- |
| **Principal focus of study** | | |
| Animal studies | | |
| Genetic or gene expression studies | | |
| Biochemistry studies or biomarkers | | |
| Endocrinology studies | | |
| Analytical methods | |  |
| Diet | |  |
| Drug therapy | |  |
| Inflammation studies | |  |
| Psychology or psychosocial studies | |  |
|  | Frailty | |
|  | Lipid profiling or metabolism studies | |
|  | Physiology | |
|  | Preclinical studies | |
|  | Prevalence | |
|  | Smoking | |
|  | Study design (including observational studies) | |
| Angiology | Adolescence/childhood | Diagnosis |
|  | Bone/bone mineral density | Proteomics |
|  | Body composition |  |
|  | Cognition studies |  |
|  | Education |  |
|  | Elite performance |  |
|  | Epidemiology |  |
|  | Health economics |  |
|  | Muscle mechanics |  |
|  | Nutrition only |  |
|  | Screening |  |
|  | Surgery |  |
|  | Vascular studies |  |
|  | Vocal function |  |
| **Disease** | | |
| Alzheimer’s | | |
| Diabetes/insulin resistance | | |
| HIV/AIDS | | |
| Obesity | | |
| Anaemia | |  |
| Anorexia | |  |
| Cancer | |  |
| Cardiac conditions | |  |
| Cirrhosis | |  |
| Dementia | |  |
| Disability | |  |
| Falls and fractures | |  |
| Muscle atrophy/disease | |  |
| Respiratory disease | |  |
|  | Cachexia | |
|  | Chronic kidney disease | |
| Allergy | Back pain | Cholesterol levels/profile |
| ALS | Cerebral palsy | Muscular dystrophy |
| Amyotrophy | Chronic disease | Not sarcopenia |
| Aortic valve replacement | COPD | Osteoporosis |
| Arthroplasty | Incontinence |  |
| Ataxia | Klotho |  |
| Bone disease | Locomotive syndrome |  |
| Charcot-Marie-Tooth disease | Musculoskeletal ambulation disability symptom complex/muscle fatigues, injury or metabolism |  |
| Cholesterol (elevated levels) | Nephrological conditions |  |
| Coeliac disease | Neurological conditions |  |
| Impaired cognition/ neurological disorders | Osteoarthritis |  |
| Critical illness | Rheumatoid arthritis |  |
| Depression | Scleroderma |  |
| Dysplasia | Swallowing difficulty |  |
| Edentulism | Weight loss |  |
| Emery–Dreyfuss dystrophy |  |  |
| End-stage renal disease/ impaired kidney function/ transplantation |  |  |
| Eye disease/ impaired visual function/ macular degeneration/ myopathy |  |  |
| Geriatric gynaecology |  |  |
| Haemophilia |  |  |
| Hearing (impaired) |  |  |
| Herniation |  |  |
| Hip or general surgery |  |  |
| Kennedy’s disease |  |  |
| King–Denborough syndrome |  |  |
| Lead poisoning |  |  |
| Liver failure |  |  |
| Malnutrition |  |  |
| Neuromuscular disease/ impaired muscle function/ musculature/ paraplegia |  |  |
| Palliative care of patients |  |  |
| Parkinson’s disease |  |  |
| Sclerosis |  |  |
| Sleep-disordered breathing |  |  |
| Stroke |  |  |
| Tuberculosis |  |  |

AIDS = acquired immunodeficiency syndrome; ALS = [amyotrophic lateral sclerosis](http://stedmansonline.com/content.aspx?id=mlrA1300005068&termtype=t); COPD = chronic obstructive pulmonary disease; HIV = human immunodeficiency virus’

**Appendix 3: Complete reference list**

1. Morley JE, Baumgartner RN, Roubenoff R, Mayer J, Nair KS. Sarcopenia. J Lab Clin Med. 2001;137:231-43.

2. Cruz-Jentoft AJ, Baeyens JP, Bauer JM, et al. Sarcopenia: European consensus on definition and diagnosis: Report of the European Working Group on Sarcopenia in Older People. Age Ageing. 2010;39:412-23.

3. Fielding RA, Vellas B, Evans WJ, et al. Sarcopenia: an undiagnosed condition in older adults. Current consensus definition: prevalence, etiology, and consequences. International working group on sarcopenia. J Am Med Dir Assoc. 2011;12:249-56.

4. Maher CG, Sherrington C, Herbert RD, Moseley AM, Elkins M. Reliability of the PEDro scale for rating quality of randomized controlled trials. Phys Ther. 2003;83:713-21.

5. Abellan van Kan G, Cesari M, Gillette-Guyonnet S, et al. Sarcopenia and cognitive impairment in elderly women: results from the EPIDOS cohort. Age Ageing. 2013;42:196-202.

6. Landi F, Cruz-Jentoft AJ, Liperoti R, et al. Sarcopenia and mortality risk in frail older persons aged 80 years and older: results from ilSIRENTE study. Age Ageing. 2013a;42:203-9.

7. Landi F, Liperoti R, Russo A, et al. Association of anorexia with sarcopenia in a community-dwelling elderly population: results from the ilSIRENTE study. Eur J Nutr. 2013b;52:1261-8.

8. Lee WJ, Liu LK, Peng LN, Lin MH, Chen LK. Comparisons of sarcopenia defined by IWGS and EWGSOP criteria among older people: results from the I-Lan longitudinal aging study. J Am Med Dir Assoc. 2013;14:528.e1-7.

9. Legrand D, Vaes B, Mathei C, Swine C, Degryse JM. The prevalence of sarcopenia in very old individuals according to the European consensus definition: insights from the BELFRAIL study. Age Ageing. 2013;42:727-34.

10. Malmstrom TK, Miller DK, Herning MM, Morley JE. Low appendicular skeletal muscle mass (ASM) with limited mobility and poor health outcomes in middle-aged African Americans. J Cachexia Sarcopenia Muscle. 2013;4:179-86.

11. McIntosh EI, Smale KB, Vallis LA. Predicting fat-free mass index and sarcopenia: a pilot study in community-dwelling older adults. Age (Dordr). 2013;35:2423-34.

12. Murphy RA, Ip EH, Zhang Q, et al. Transition to Sarcopenia and Determinants of Transitions in Older Adults: A Population-Based Study. J Gerontol A Biol Sci Med Sci. 2013 Sept 7:10.1093/gerona/glt131.

13. Patel HP, Syddall HE, Jameson K, et al. Prevalence of sarcopenia in community-dwelling older people in the UK using the European Working Group on Sarcopenia in Older People (EWGSOP) definition: findings from the Hertfordshire Cohort Study (HCS). Age Ageing. 2013;42:378-84.

14. Patil R, Uusi-Rasi K, Pasanen M, Kannus P, Karinkanta S, Sievanen H. Sarcopenia and osteopenia among 70-80-year-old home-dwelling Finnish women: prevalence and association with functional performance. Osteoporos Int. 2013;24:787-96.

15. Sanada K, Iemitsu M, Murakami H, et al. Adverse effects of coexistence of sarcopenia and metabolic syndrome in Japanese women. Eur J Clin Nutr. 2012;66:1093-8.

16. Tanimoto Y, Watanabe M, Sun W, et al. Association between sarcopenia and higher-level functional capacity in daily living in community-dwelling elderly subjects in Japan. Arch Gerontol Geriatr. 2012;55:e9-13.

17. Verschueren S, Gielen E, O'Neill TW, et al. Sarcopenia and its relationship with bone mineral density in middle-aged and elderly European men. Osteoporos Int. 2013;24:87-98.

18. Volpato S, Bianchi L, Cherubini A, et al. Prevalence and clinical correlates of sarcopenia in community-dwelling older people: application of the EWGSOP definition and diagnostic algorithm. J Gerontol A Biol Sci Med Sci. 2014;69:438-46.

19. Yamada M, Nishiguchi S, Fukutani N, et al. Prevalence of sarcopenia in community-dwelling Japanese older adults. J Am Med Dir Assoc. 2013;14:911-5.

20. Bastiaanse LP, Hilgenkamp TI, Echteld MA, Evenhuis HM. Prevalence and associated factors of sarcopenia in older adults with intellectual disabilities. Res Dev Disabil. 2012;33:2004-12.

21. Landi F, Liperoti R, Fusco D, et al. Prevalence and risk factors of sarcopenia among nursing home older residents. J Gerontol A Biol Sci Med Sci. 2012;67:48-55.

22. Gariballa S, Alessa A. Sarcopenia: prevalence and prognostic significance in hospitalized patients. Clin Nutr. 2013;32:772-6.

23. Binder EF, Yarasheski KE, Steger-May K, et al. Effects of progressive resistance training on body composition in frail older adults: results of a randomized, controlled trial. J Gerontol A Biol Sci Med Sci. 2005;60:1425-31.

24. Bonnefoy M, Cornu C, Normand S, et al. The effects of exercise and protein-energy supplements on body composition and muscle function in frail elderly individuals: a long-term controlled randomised study. Br J Nutr. 2003;89:731-9.

25. Bunout D, Barrera G, de la Maza P, et al. The impact of nutritional supplementation and resistance training on the health functioning of free-living Chilean elders: results of 18 months of follow-up. J Nutr. 2001;131:2441s-6s.

26. Goodpaster BH, Chomentowski P, Ward BK, et al. Effects of physical activity on strength and skeletal muscle fat infiltration in older adults: a randomized controlled trial. J Appl Physiol (1985). 2008;105:1498-503.

27. Kemmler W, von Stengel S, Engelke K, Haberle L, Mayhew JL, Kalender WA. Exercise, body composition, and functional ability: a randomized controlled trial. Am J Prev Med. 2010;38:279-87.

28. Rydwik E, Lammes E, Frandin K, Akner G. Effects of a physical and nutritional intervention program for frail elderly people over age 75. A randomized controlled pilot treatment trial. Aging Clin Exp Res. 2008;20:159-70.

29. Suetta C, Andersen JL, Dalgas U, et al. Resistance training induces qualitative changes in muscle morphology, muscle architecture, and muscle function in elderly postoperative patients. J Appl Physiol (1985). 2008;105:180-6.

30. Chale A, Cloutier GJ, Hau C, Phillips EM, Dallal GE, Fielding RA. Efficacy of whey protein supplementation on resistance exercise-induced changes in lean mass, muscle strength, and physical function in mobility-limited older adults. J Gerontol A Biol Sci Med Sci. 2013;68:682-90.

31. Cornish SM, Chilibeck PD. Alpha-linolenic acid supplementation and resistance training in older adults. Appl Physiol Nutr Metab. 2009;34:49-59.

32. Deutz NE, Pereira SL, Hays NP, et al. Effect of beta-hydroxy-beta-methylbutyrate (HMB) on lean body mass during 10 days of bed rest in older adults. Clin Nutr. 2013;32:704-12.

33. Dillon EL, Sheffield-Moore M, Paddon-Jones D, et al. Amino acid supplementation increases lean body mass, basal muscle protein synthesis, and insulin-like growth factor-I expression in older women. J Clin Endocrinol Metab. 2009;94:1630-7.

34. Flakoll P, Sharp R, Baier S, Levenhagen D, Carr C, Nissen S. Effect of beta-hydroxy-beta-methylbutyrate, arginine, and lysine supplementation on strength, functionality, body composition, and protein metabolism in elderly women. Nutrition. 2004;20:445-51.

35. Kim HK, Suzuki T, Saito K, et al. Effects of exercise and amino acid supplementation on body composition and physical function in community-dwelling elderly Japanese sarcopenic women: a randomized controlled trial. J Am Geriatr Soc. 2012;60:16-23.

36. Stout JR, Smith-Ryan AE, Fukuda DH, et al. Effect of calcium beta-hydroxy-beta-methylbutyrate (CaHMB) with and without resistance training in men and women 65+yrs: a randomized, double-blind pilot trial. Exp Gerontol. 2013;48:1303-10.

37. Tieland M, Dirks ML, van der Zwaluw N, et al. Protein supplementation increases muscle mass gain during prolonged resistance-type exercise training in frail elderly people: a randomized, double-blind, placebo-controlled trial. J Am Med Dir Assoc. 2012a;13:713-9.

38. Tieland M, van de Rest O, Dirks ML, et al. Protein supplementation improves physical performance in frail elderly people: a randomized, double-blind, placebo-controlled trial. J Am Med Dir Assoc. 2012b;13:720-6.

39. Vukovich MD, Stubbs NB, Bohlken RM. Body composition in 70-year-old adults responds to dietary beta-hydroxy-beta-methylbutyrate similarly to that of young adults. J Nutr. 2001;131:2049-52.

40. Bauer J, Biolo G, Cederholm T, et al. Evidence-based recommendations for optimal dietary protein intake in older people: a position paper from the PROT-AGE Study Group. J Am Med Dir Assoc. 2013;14:542-59.
